# Conditioning on parental mating types can reduce necessary assumptions for Mendelian randomization

**DOI:** 10.3389/fgene.2023.1014014

**Published:** 2023-03-06

**Authors:** Keisuke Ejima, Nianjun Liu, Luis Miguel Mestre, Gustavo de los Campos, David B. Allison

**Affiliations:** ^1^ Department of Epidemiology and Biostatistics, Indiana University School of Public Health-Bloomington, Bloomington, IN, United States; ^2^ Lee Kong Chian School of Medicine, Nanyang Technological University, Singapore, Singapore; ^3^ Department of Global Health Policy, Graduate School of Medicine, The University of Tokyo, Tokyo, Japan; ^4^ Department of Epidemiology and Biostatistics, Michigan State University, East Lansing, MI, United States; ^5^ Department of Statistics and Probability, Michigan State University, East Lansing, MI, United States; ^6^ Institute for Quantitative Health Science and Engineering, Michigan State University, East Lansing, MI, United States

**Keywords:** Mendelian randomization, genetic epidemiology, causal inference, study design, linkage disequilibrium

## Abstract

Mendelian randomization (MR) has become a common tool used in epidemiological studies. However, when confounding variables are correlated with the instrumental variable (in this case, a genetic/variant/marker), the estimation can remain biased even with MR. We propose conditioning on parental mating types (a function of parental genotypes) in MR to eliminate the need for one set of assumptions, thereby plausibly reducing such bias. We illustrate a situation in which the instrumental variable and confounding variables are correlated using two unlinked diallelic genetic loci: one, an instrumental variable and the other, a confounding variable. Assortative mating or population admixture can create an association between the two unlinked loci, which can violate one of the necessary assumptions for MR. We simulated datasets involving assortative mating and population admixture and analyzed them using three different methods: 1) conventional MR, 2) MR conditioning on parental genotypes, and 3) MR conditioning on parental mating types. We demonstrated that conventional MR leads to type I error rate inflation and biased estimates for cases with assortative mating or population admixtures. In the presence of non-additive effects, MR with an adjustment for parental genotypes only partially reduced the type I error rate inflation and bias. In contrast, conditioning on parental mating types in MR eliminated the type I error inflation and bias under these circumstances. Conditioning on parental mating types is a useful strategy to reduce the burden of assumptions and the potential bias in MR when the correlation between the instrument variable and confounders is due to assortative mating or population stratification but not linkage.

## Introduction

Randomized experiments, often called randomized controlled trials, are the gold standard for drawing causal inferences. In randomized experiments, observational units (e.g., subjects) are randomly assigned to different levels of the variable being used to assess the causal effect, e.g., the treatment. The randomization process eliminates the influence of potential confounding variables on the exposure variable (e.g., treatment or control). Therefore, we can conclude that the observed difference in outcomes between groups in randomized controlled trials is purely caused by the treatment (barring stochastic variations). However, randomized experiments are not always ethical, feasible, or practical (Sanson-Fisher et al., 2007).

Observational studies do not always yield unbiased estimates of effects because of their lack of random assignment. Of the multiple limitations that these studies have, herein, we will only consider the bias due to confounding.

**FIGURE 1:**
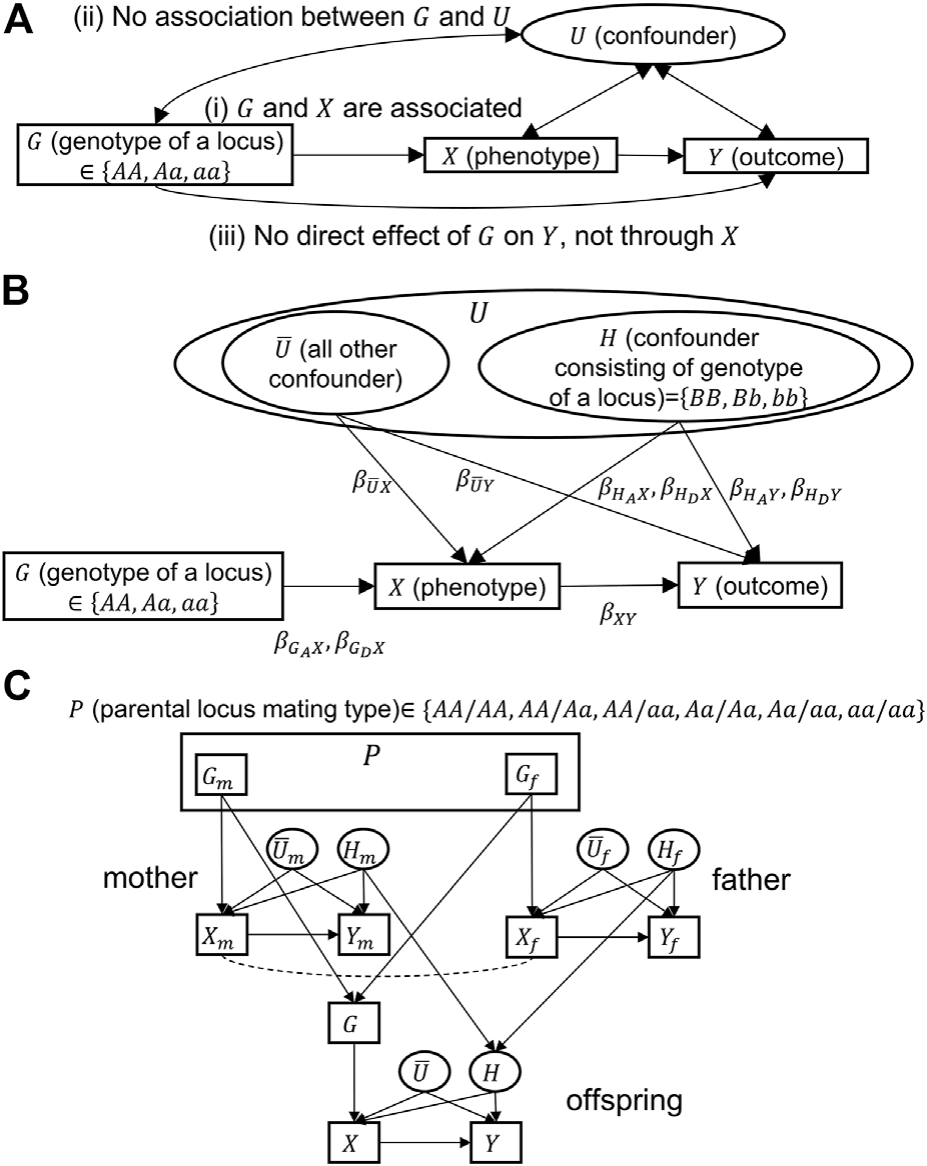
Causal models for Mendelian randomization. Directional and bidirectional arrows correspond to causal and associational relationships, respectively. β s are regression coefficients. Variables in rectangles and ovals correspond to measurable or unmeasurable variables, respectively. **(A)** Generalized model for Mendelian randomization (MR) with three assumptions. **(B)** Explicit causal model separating confounding variables, U , into the variable consisting of unlinked heritable variants in the nuclear genome, H , and all other confounding variables, $\bar{U}$ . **(C)** Explicit causal model for the father–mother–offspring trio. The parental mating type, P , is the combination of parental genotypes ( $G_{m}$ and $G_{f}$ ), which takes one of the six possible values. The dotted line connecting $X_{m}$ and $X_{f}$ implies assortative mating of these variables.

To mitigate confounding, researchers often include potential confounders in analyses as covariates in regression models or stratify analyses by confounders. Figure 1A depicts a general causal model with an exposure variable ( X ), an outcome ( Y ), a confounder ( U ), and a genetic marker ( G ), where U is associated with both X and Y and G determines X . The variables X , Y , and U are assumed to be continuous. Causal effects and associations are represented by directional and bidirectional arrows, respectively. If U is observable and is included in the model, the estimate of the effect of X on Y will be unbiased, provided the estimation method does not induce a bias. However, the confounder ( U ) is not always measurable or known. If U is a set of confounders of the relationship between X and Y and is not appropriately accounted for in the analysis, the estimator of the regression coefficient of Y on X will be biased.

Mendelian randomization (MR) was proposed to address the issue of unmeasured confounders in observational studies (Smith and Ebrahim, 2003; Boutwell and Adams, 2020; Sanderson et al., 2022). MR uses genotypic ( G ) data from loci that affect the exposure variable ( X ), do not have a direct effect on the outcome, and are uncorrelated with potential confounders. The most commonly used process of estimation is as follows: 1) X is regressed on G to obtain the predicted value of X , $\hat{X}$ ; 2) Y is regressed on $\hat{X}$ , and then, the estimated coefficient is an unbiased estimator of the effect of X on Y under some assumptions. As a simple and robust approach for causal inference, MR has become common in epidemiological studies during the last few decades.

However, MR rests on three assumptions (Emdin et al., 2017): “1) the genetic variant is associated with the risk factor; 2) the genetic variant is not associated with confounders; and 3) the genetic variant influences the outcome only through the risk factor.” In Figure 1A, these assumptions correspond to the following: 1) G and X are associated, 2) there is no association between G and U , and 3) there is no direct effect of G on Y , not through X . If any of the aforementioned three assumptions are violated, the estimated effect is not guaranteed to be unbiased.

Unfortunately, the violation of assumptions, especially the violation of assumption (2), is quite plausible: the genotype ( G ) can be associated with confounders ( U ). Even without a direct effect of G on U (or *vice versa*), assortative mating and population stratification can yield associations between them, which violate assumption (2). Furthermore, it is hard to verify this assumption because U includes unmeasurable variables: “The second and third assumptions, however, cannot be empirically proven and require both judgment by the investigators and the performance of various sensitivity analyses” (Emdin et al., 2017). This paper proposes conditioning on parental mating types (defined as a combination of genotypes of parents at a locus used as an instrumental variable (Allison, 1997)) in MR to eliminate the bias in conventional MR, when there is correlation between the instrumental variable and confounding variables. This means that our approach obviates the need for one of the three necessary assumptions in MR.

This paper consists of two parts. First, we demonstrate that the estimation using conventional MR (without conditioning on parental mating types) could lead to biased estimates, when there is a correlation between the instrumental variable and confounding variables due to assortative mating or population stratification. Second, we propose the use of parental mating types in conventional MR and to assess the utility of this approach.

## Materials and methods

### Mechanisms violating assumptions for MR: Assortative mating and population stratification

First, we define the variables, parameters, and error terms used in the simulation and analyses (summarized in Table 1), with explicit mathematical expressions and causal mechanisms. There are six variables: X , Y , U , G , H , and P . X , Y , and $\bar{U}$ are an exposure variable, an outcome variable, and a confounder, respectively, and all are quantitative traits (thus, continuous variables), such as weight and height. G and H are genotypes defined by SNPs; thus, they are one of the three statuses: $\left\{AA,Aa,aa\right\}$ for G and $\left\{BB,Bb,bb\right\}$ for H , respectively; $f_{A}\left(*\right)$ and $f_{D}\left(*\right)$ are functions to calculate the additive and dominance effect of a genotype, respectively ( $f_{A}$ counts the number of A [or B ] alleles for the genotype; $f_{D}$ is 1 for a heterozygote and 0 for a homozygote). P is the parental mating type, a combination of genotypes of parents at a locus used as an instrumental variable, and one of the six statuses: $\left\{AA/AA,AA/Aa,AA/aa,Aa/Aa,Aa/aa,aa/aa\right\}$ . We introduce five indicator functions to compute the genetic effect of parental mating types, $I_{AA/AA}\left(P\right),I_{AA/Aa}\left(P\right),I_{AA/aa}\left(P\right),I_{Aa/Aa}\left(P\right),I_{Aa/aa}\left(P\right)$ , where the function is 1 if P is the same as the subscript of the function and otherwise, 0. The effect of a variable M on a variable N (i.e., the difference in N due to a single unit increase in M ) is represented by $\beta_{MN}$ . It should be noted that the additive effect and the dominance effect of a genotype M on a variable N are represented as $\beta_{M_{A}N}$ (i.e., difference in N by substituting allele A [or B ] for allele a [or b ]) and $\beta_{M_{D}N}$ (i.e., deviance from the average of genotypic values of the two homozygotes), respectively. Furthermore, there are five coefficients to represent the effect of parental mating type P on a variable M using the parental mating type aa/aa as a reference group. Thus, for example, $\beta_{AA/AAM}$ is the unit increase in M for the parental mating type AA/AA compared to the increase in the parental mating type aa/aa . Estimated regression coefficients are distinguished from the causal effect using the following: ${\hat{\beta }}_{MN}$ is the regression coefficient estimated by regressing N on M .

**TABLE 1:** Summary of variables, functions, and intercepts in regression models.

Parameter	Description
X	Exposure variable
Y	Outcome
G	Genotype of a locus with effects on X $\left(G\in \left\{AA,Aa,aa\right\}\right)$
H	Confounder consisting of a genotype of a locus with effects on X and Y $\left(H\in \left\{BB,Bb,bb\right\}\right)$
$\bar{U}$	All other (non-genetic) confounders (with effects on X and Y )
P	Parental mating type on $G\;\left(P\in \left\{AA/AA,AA/Aa,AA/aa,Aa/Aa,Aa/aa,aa/aa\right\}\right)$

Function for genetic effects	Description
$f_{A}\left(M\right)$	Function to compute the additive effect of genotype M (counting the number of A or B )
$f_{D}\left(M\right)$	Function to compute the dominance effect of genotype M (1 for a heterozygote and 0 for a homozygote)
$I_{AA/AA}\left(P\right)$	Indicator function (1 for the parental mating type AA/AA and 0 for the other)
$I_{AA/Aa}\left(P\right)$	Indicator function (1 for the parental mating type AA/Aa and 0 for the other)
$I_{AA/aa}\left(P\right)$	Indicator function (1 for the parental mating type AA/aa and 0 for the other)
$I_{Aa/Aa}\left(P\right)$	Indicator function (1 for the parental mating type Aa/Aa and 0 for the other)
$I_{Aa/aa}\left(P\right)$	Indicator function (1 for the parental mating type Aa/aa and 0 for the other)
$I_{aa/aa}\left(P\right)$	Indicator function (1 for the parental mating type aa/aa and 0 for the other)

Intercept in the regression model	Description
$\beta_{1}$	Genotypic value of aa on X
$\beta_{2}$	Genotypic value of bb on Y when both X and $\bar{U}$ are zero

Figure 1B is a causal model in which the confounding variable set, U , is separated into two sets of variables: one set includes a confounding variable consisting of a genotype on a single biallelic locus (*H*) with two alleles B and b , and the second set $\bar{U}$ consists of all other confounders. We note that in our scenario, H and the genotype on the biallelic locus, used as an instrumental variable ( G ), are unlinked. However, G and H could be correlated (i.e., non-linkage disequilibrium), which would violate assumption (2). The following describes two situations, assortative mating and population stratification, which can cause such a non-linkage disequilibrium.

#### Situation 1: Assortative mating

In human and other animal populations, the choice of a mate does not plausibly occur at random. One may be more likely to mate with another who has specific phenotypes, resulting in non-random or assortative mating (Anonymous, 1903). For example, assortative mating for body mass index (BMI) or body fatness (i.e., individuals with a high BMI or body fatness are more likely to mate with one another, as are individuals with low BMI or body fatness) is widely observed (Allison et al., 1996; Silventoinen et al., 2003; Jackson et al., 2007). We modeled assortative mating as being dependent on the exposure variable X . Mothers and fathers are separately sorted by X , and they are paired according to the order. For this purpose, each parent’s genotype, exposure, outcome, and confounders are explicitly modeled. Variables are given with one of the two subscripts, m or f , for either the mother or the father (variables without these subscripts are for an offspring). The model is summarized in Figure 1C.

Briefly, the correlation between G and H is explained as follows: Assortative mating on X (i.e., $X_{m}$ and $X_{f}$ ) induces associations between $G_{m}$ and $H_{f}$ and $G_{f}$ and $H_{m}$ , which result in an association between G and H , thus violating the MR assumption (2).

#### Situation 2: Population stratification

Population stratification occurs and can create genotype–phenotype associations in the absence of linkage or a causal effect of the specific genotype on the specific phenotype, when a population consists of multiple subpopulations (Freedman et al., 2004) and some subpopulations have different allele frequencies and phenotypic distributions. By using the framework given in Figure 1C without assortative mating, we assume two different subpopulations. Therefore, within each subpopulation, three assumptions are held for conventional MR. The difference between the two populations is that they have different allele frequencies. If data from the two subpopulations were analyzed as a single population without accounting for the population substructure, they would yield a spurious association between G and H (because all parental loci [ $G_{m}$ , $H_{m}$ , $G_{f},$ and $H_{f}$ ] are associated), which violates the MR assumption (2).

### Correcting the bias in MR: Conditioning on parental mating types

Assuming the aforementioned two situations, the conventional MR estimation procedure can lead to biased estimates because MR assumption (2) is violated. To eliminate the bias, we propose conditioning on the parental mating type P , which is a combination of parental genotypes used for the instrumental variable in MR. The rationale for using P is that both $G_{m}$ and $G_{f}$ are located on open (i.e., d connected) paths between genotypes G and H in both situations 1 and 2, and conditioning on P blocks the path. We also follow the approach of using parental *genotypes* instead of *mating types*, as proposed by Hartwig et al. (2018), which is another reference method.

In the following, we show details of three methods: conventional MR, MR conditioning on parental genotypes [a method proposed by Hartwig et al. (2018)], and MR conditioning on parental mating types (which we propose in this study). It should be noted that unmeasurable variables (variables in ovals, given in Figure 1C) do not appear in any of the analyses.

#### Conventional MR

1) Conventional MR uses the following model: $X=\beta_{1}+\beta_{G_{A}X}f_{A}\left(G\right)+\beta_{G_{D}X}f_{D}\left(G\right)+\varepsilon_{X}$ , where $\varepsilon_{X}$ is an error term. Therefore, the auxiliary regression of X on $f_{A}\left(G\right)$ and $f_{D}\left(G\right)$ is performed to obtain the estimated value of X (= $\hat{X}$ ): $\hat{X}={\hat{\beta }}_{1}+{\hat{\beta }}_{G_{A}X}f_{A}\left(G\right)+{\hat{\beta }}_{G_{D}X}f_{D}\left(G\right)$ .
2) Then, the regression of Y on $\hat{X}$ is conducted by assuming the following model with an error term $\varepsilon_{Y}$ : $Y=\beta_{2}+\beta_{XY}\hat{X}+\varepsilon_{Y}$ .

#### MR conditioning on parental genotypes

To correct for the bias in MR, Hartwig et al. (2018) proposed conditioning on parental genotypes. The analysis proceeds as follows:

1) MR conditioning on parental genotypes uses the following model: $X=\beta_{1}+\beta_{G_{A}X}f_{A}\left(G\right)+\beta_{G_{D}X}f_{D}\left(G\right)+\beta_{G_{mA}X}f_{A}\left(G_{m}\right)+\beta_{G_{mD}X}f_{D}\left(G_{m}\right)+\beta_{G_{fA}X}f_{A}\left(G_{f}\right)+\beta_{G_{fD}X}f_{D}\left(G_{f}\right)+\varepsilon_{X}$ . Therefore, the auxiliary regression of X on $f_{A}\left(G\right)$ and $f_{D}\left(G\right)$ conditioning on $f_{A}\left(G_{m}\right)$ , $f_{D}\left(G_{m}\right)$ , $f_{A}\left(G_{f}\right)$ , and $f_{D}\left(G_{f}\right)$ are performed to obtain the estimated value of X (= $\hat{X}$ ): $\hat{X}={\hat{\beta }}_{1}+{\hat{\beta }}_{G_{A}X}f_{A}\left(G\right)+{\hat{\beta }}_{G_{D}X}f_{D}\left(G\right)+{\hat{\beta }}_{G_{mA}X}f_{A}\left(G_{m}\right)+{\hat{\beta }}_{G_{mD}X}f_{D}\left(G_{m}\right)+{\hat{\beta }}_{G_{fA}X}f_{A}\left(G_{f}\right)+{\hat{\beta }}_{G_{fD}X}f_{D}\left(G_{f}\right)$ .
2) The regression of Y on $\hat{X}$ is conducted assuming the following model: $Y=\beta_{2}+\beta_{XY}\hat{X}+\beta_{G_{mA}Y}f_{A}\left(G_{m}\right)+\beta_{G_{mD}Y}f_{D}\left(G_{m}\right)+\beta_{G_{fA}Y}f_{A}\left(G_{f}\right)+\beta_{G_{fD}Y}f_{D}\left(G_{f}\right)+\varepsilon_{Y}$ .

#### MR conditioning on parental mating types

Hartwig et al. (2018) assumed an additive model and, thus, used a *parental genotype* as an instrumental variable. However, if the effect of the parental genotype on an offspring’s phenotype is non-additive, using a parental mating type, i.e., a combination of parental genotypes taking one of the six possible values (Figure 1C), is more appropriate. The corresponding analysis proceeds as follows:

1) MR conditioning on parental mating types uses the following model: $X=\beta_{1}+\beta_{G_{A}X}f_{A}\left(G\right)+\beta_{G_{D}X}f_{D}\left(G\right)+\beta_{AA/AAX}I_{AA/AA}\left(P\right)+\beta_{AA/AaX}I_{AA/Aa}\left(P\right)+\beta_{AA/aaX}\;I_{AA/aa}\left(P\right),+\beta_{Aa/AaX}I_{Aa/Aa}\left(P\right)+\beta_{Aa/aaX}\;I_{Aa/aa}\left(P\right)+\varepsilon_{X}$ . Therefore, the auxiliary regression of X on $G_{1}$ conditioning on the parental mating type P is performed to obtain the estimated value of X (= $\hat{X}$ ): $\hat{X}={\hat{\beta }}_{1}+{\hat{\beta }}_{G_{A}X}f_{A}\left(G\right)+{\hat{\beta }}_{G_{D}X}f_{D}\left(G\right)+{\hat{\beta }}_{AA/AAX}I_{AA/AA}\left(P\right)+{\hat{\beta }}_{AA/AaX}I_{AA/Aa}\left(P\right)+{\hat{\beta }}_{AA/aaX}\;I_{AA/aa}\left(P\right),+{\hat{\beta }}_{Aa/AaX}I_{Aa/Aa}\left(P\right)+{\hat{\beta }}_{Aa/aaX}\;I_{Aa/aa}\left(P\right).$

2) The regression of Y on $\hat{X}$ is conducted by assuming the following model: $Y=\beta_{2}+\beta_{XY}\hat{X}+{\hat{\beta }}_{AA/AAX}I_{AA/AA}\left(P\right)+{\hat{\beta }}_{AA/AaX}I_{AA/Aa}\left(P\right)+{\hat{\beta }}_{AA/aaX}\;I_{AA/aa}\left(P\right),+{\hat{\beta }}_{Aa/AaX}I_{Aa/Aa}\left(P\right)+{\hat{\beta }}_{Aa/aaX}\;I_{Aa/aa}\left(P\right)+\varepsilon_{Y}$ .

### Simulations

To demonstrate the potential bias when conventional MR is used due to the violation of the MR assumption (2) and the utility of using parental mating types to eliminate the bias, we performed simulations considering assortative mating and population stratification.

For the simulation of each situation, we created data for 1,000 trio (father–mother–offspring) families (500 trios each for the population for situation 2) for a single simulation and performed three different analyses on each dataset. We repeated the process 1,000 times for each parameter setting. The type I error rate (when $\beta_{XY}=0$ ) is defined as the proportion of simulations in which the estimated association between X and Y is statistically significant (false-positive finding). The bias in the estimated coefficient $E\left[{\hat{\beta }}_{XY}-\beta_{XY}\right]$ is also assessed when $\beta_{XY}>0$ . The coefficient $\beta_{XY}$ was set as 1.0 for bias assessment. The sensitivity of the type I error rate and the bias on the magnitude of the violation of MR assumption (2) were assessed by varying the parameters. The significance level was set as 0.05. The process for generating data and analyses are described in the next section.

#### Simulation 1: Assortative mating

The following is a step-by-step protocol and parameter setting for the simulation:

1) Allele frequencies of A and B are 10% for each: $Prob\left(A\right)=Prob\left(B\right)=0.1,Prob\left(a\right)=Prob\left(b\right)=0.9$ . Each parent’s genotypes ( $G_{m}$ , $H_{m}$ , $G_{f}$ , and $H_{f}$ ) are determined assuming the Hardy–Weinberg equilibrium (Hardy, 1908). It should be noted that G and H are independent.
2) The confounding variables for parents, ${\bar{U}}_{m}$ and ${\bar{U}}_{f}$ , are determined, which follow a bivariate normal distribution: $N\left(0,0.1\right)$ .
3) The exposure variables of parents, $X_{m}$ and $X_{f}$ , are determined by their genotype and confounding variable: $X_{m}=\beta_{1}+\beta_{G_{A}X}f_{A}\left(G_{m}\right)+\beta_{G_{D}X}f_{D}\left(G_{m}\right)+\beta_{\bar{U}X}{\bar{U}}_{m}+\varepsilon_{X}$ , where $\varepsilon_{X}∼N\left(0,0.1\right)$ . $\beta_{1}$ is interpreted as the genotypic effect of the genotype aa on X . $X_{f}$ is determined in the same way as $X_{m}$ .
4) The outcome of parents, $Y_{m}$ and $Y_{f}$ , are determined by their exposure, genotype, and confounding variable: $Y_{m}=\beta_{2}+\beta_{XY}X_{m}+\beta_{H_{A}Y}f_{A}\left(H_{m}\right)+\beta_{H_{D}Y}f_{D}\left(H_{m}\right)+\beta_{\bar{U}Y}{\bar{U}}_{m}+\varepsilon_{Y}$ , where $\varepsilon_{Y}∼N\left(0,0.1\right)$ . $\beta_{2}$ is interpreted as the genotypic effect of the genotype bb on Y, when both X and $\bar{U}$ are zero. $Y_{f}$ is determined in the same way as $Y_{m}$ .
5) Proportion p is selected from paternal and maternal populations. In the selected population, both parents are sorted separately by the exposure $X_{m}$ or $X_{f}$ and are paired according to the order of $X_{m}$ and $X_{f}$ . Unselected parents (1- p ) are randomly coupled regardless of the values of X and Y .
6) The genotype of the offspring, G and H , are determined by randomly selecting an allele from each parent.
7) The exposure, X , and the outcome, Y , of the offspring are determined by following the same process as for the parents (see 3 and 4).

The sensitivity of the type I error rate and bias was assessed by changing p from 0.0 to 0.8. All effects from $\bar{U}$ to X and Y are assumed to be 1. For genetic effects, we assumed that there is no additive effect ( $\beta_{G_{A}X}=\beta_{H_{A}X}=\beta_{H_{A}Y}=0$ ), but there is a strong dominance effect ( $\beta_{G_{D}X}=\beta_{H_{D}X}=\beta_{H_{D}Y}=1$ ) of G and H on any associated variables.

#### Simulation 2: Population stratification

The simulation setting for simulation 2 is similar to simulation 1 save for a couple of differences: 1) no assortative mating and 2) we assume two populations (i.e., subpopulation 1 and subpopulation 2) with different allele frequencies. Allele frequencies of A and B for subpopulation 1 are 10% each. Otherwise, all simulation settings, including parameter settings, are the same as those in simulation 1. The source of the violation of MR assumption (2) is different allele frequencies. To demonstrate the sensitivity of the type I error rate and bias on the magnitude of the violation of MR assumption (2), allele frequencies of A and B for subpopulation 2 were varied from 10% to 90%. All simulations and analyses were performed using statistical computing software R (version 3.6.1).

## Results

**FIGURE 2:**
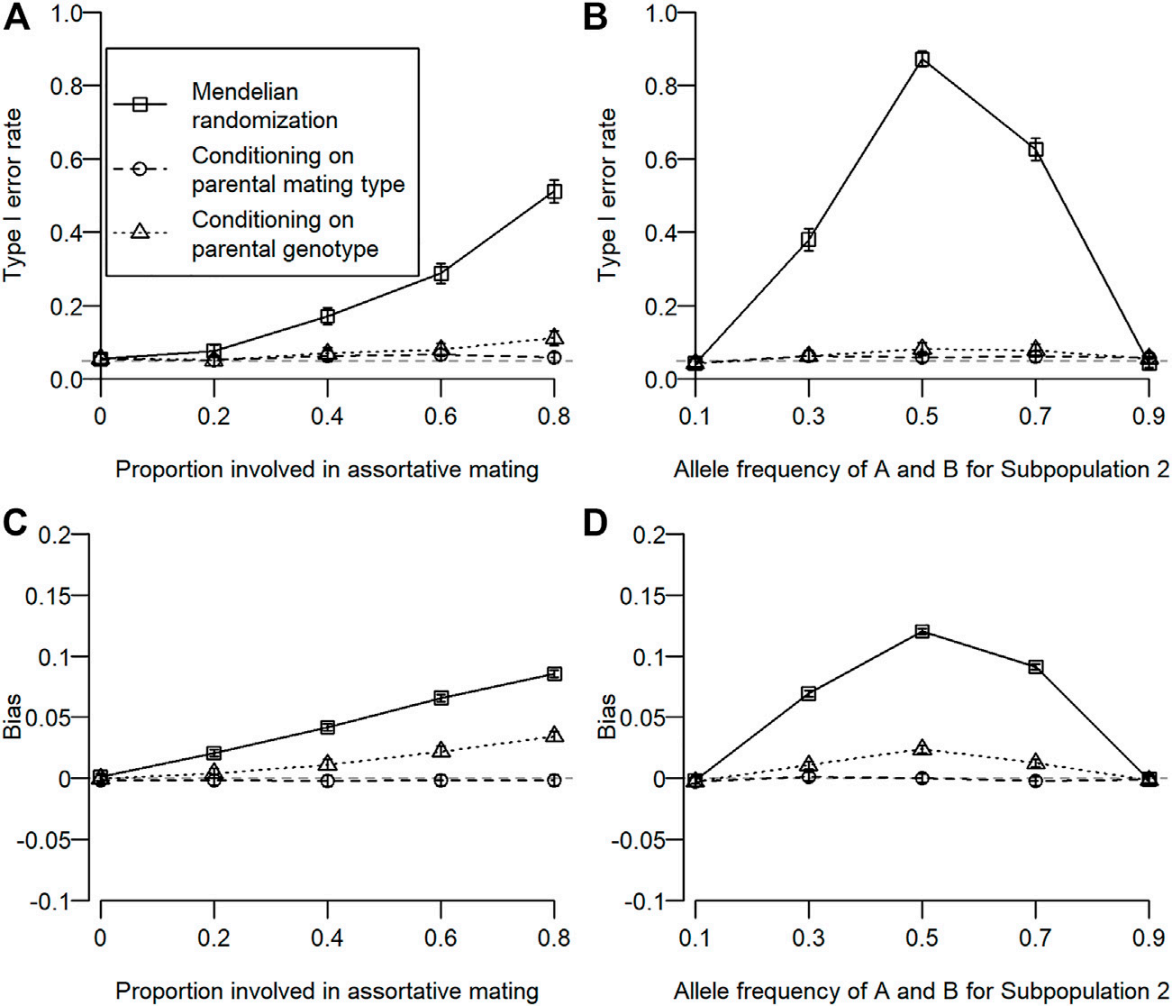
Type I error rate and bias of estimated coefficients for three different types of MR. Open squares, open circles, and open triangles correspond to conventional MR, MR conditioning on parental mating types, and MR conditioning on parental genotypes, respectively. For simulation 1, the proportion of the population involved in assortative mating was changed from 0 to 0.8. For simulation 2, allele frequencies of A and B for subpopulation 2 were varied from 10% to 90%. (**A, B)** Type I error rates for simulations 1 and 2. Gray dotted lines are significance levels (= 0.05). (**C, D)** Bias in the estimated regression coefficient of an offspring’s outcome on exposure ( ${\hat{\beta }}_{XY}-\beta_{XY}$ ) for simulations 1 and 2.

The type I error rate for simulation 1 is shown in Figure 2A. Type I error inflation was observed for conventional MR and MR conditioning on parental genotypes, and it increased as the proportion involved in assortative mating increased. Type I error inflation was not observed for MR conditioning on parental mating types. Type I error inflation was mitigated by conditioning on parental genotypes to some extent, which still remained. The type I error rate for simulation 2 is shown in Figure 2B. Type I error inflation was observed for both conventional MR and MR conditioning on parental genotypes but not for MR conditioning on parental mating types. As shown in simulation 1, conditioning on parental genotypes reduced but did not eliminate type I error rate inflation. Interestingly, we observed a large type I error inflation when allele frequencies for the subpopulation were intermediate (0.5). This is because we assumed that homozygous genotypes (i.e., AA,aa and BB,bb ) have the same effect on phenotypes ( X and Y ).

The bias of the estimated coefficient is shown in Figures 2C, D. We observed similar results for the bias in estimation as in type I error rates. When type I error rate inflation was observed, a statistically significant bias was also observed, and magnitudes of type I error rate inflation and absolute bias were positively associated.

## Discussion

MR has become a common approach for causal inference in epidemiology, as genetic data become more accessible owing to fast and efficient DNA sequencing technology and as journals and funding bodies encourage data sharing (Levey et al., 2009; Bloom et al., 2014; Loder and Groves, 2015). However, as for most epidemiological approaches, MR has essential assumptions we need to check before performing analysis. Among them, the assumption of no association between genetic variants used in MR and confounders [MR assumption (2)] could be violated or is difficult to check in practice. First, we demonstrated that MR produces inflation in type I error rates and a biased estimation in realistic settings where the assumption is violated. We introduced two plausible situations: assortative mating and population stratification. The sensitivity of type I error rates and estimation bias was assessed by changing parameters relevant to the violation of the MR assumption. As expected, we observed type I error inflation and estimation bias in these realistic settings when conventional MR was used, and such inflations and biases worsened as violations became more severe. They were mitigated by conditioning on parental genotypes to some extent; however, type I error inflation remained. Second, we proposed the use of parental mating types for a valid association inference for these two situations. We successfully confirmed that conditioning on parental mating types solves the problem in both situations.

We noted that we are not the first to propose the idea of considering parental genetic information in an epidemiological study. The idea was originally proposed in testing for linkages in the presence of associations (Allison, 1997). Redden et al. suggested using parental mating types in the inference of genotype–phenotype associations (Redden and Allison, 2006). Later, Liu et al. (2015) extended the idea to testing causal effects of a fetal drive. In this work, they showed the relationship between this idea and MR. In MR, the genetic variant needs to be a causal variant. However, it may be difficult to verify this assumption in practice, if not impossible. Conditioning on parental mating types is one way to identify causal genetic variants, thus relaxing assumptions, specifically assumption (2) of MR (resulting in the strengthening of MR). In the context of MR, Hartwig et al. proposed using parental genotypes in the case of assortative mating, which violates MR assumption (3) (Hartwig et al., 2018). They proposed two methods to integrate parental genotypes in MR analyses. The first method is to adjust conventional MR by parental allele scores, which we used in this study. The second method is to use parental non-transmitted allele scores and the offspring allele score as instrumental variables of parental and offspring exposure variables. They demonstrated that both methods provide unbiased estimates of the exposure–outcome association and avoid type I error inflation even under strong assortative mating conditions. The difference between the study by Hartwig et al. and ours is that we assumed that the locus influencing the outcome ( H ) also influences the exposure ( X ). Therefore, their model is considered a special case of ours. Although Hartwig et al. (2018) concluded that only cross-trait assortative mating (between X and Y ) yields a bias, we found that same-trait assortative mating (between X s or between Y s) can also yield a bias due to the heritable confounding variable ( H ). Furthermore, we found that conditioning of parental genotypes is not enough to control the bias if effects of alleles on phenotypes are non-additive. In our previous work (Liu et al., 2015), we indicated that random mating is not assumed with conditioning on parental mating types. We also explained that it is necessary to condition on parental mating types to achieve randomization, which is the basis for causal inference. Further insights into the rationale for this or other ways of expressing fundamental ideas can be found in the study by Pearl et al. (2016).

We list a few limitations of our approach. One apparent limitation is the data availability. Most genetic epidemiological research studies do not have (or is not designed to collect) parental genetic data (i.e., mother–father–offspring). However, because family trio data collection is considered to be a powerful tool for identifying rare diseases, even outside the context of MR, and owing to technological advancements in gene sequencing, the collection of family trio data may become more common (Infante-Rivard et al., 2009). In a recent study, Young et al. proposed imputing parental genotypes to reduce biases in GWA studies (Young et al., 2022). The imputation strategy presented in this study provides an opportunity to implement methods we proposed here for MR in situations where parental genotypes are not directly available. In this work, we propose that conditioning on parental genetic mating types can reduce assumptions needed for MR. We illustrate this key principle using a simulation study involving one locus with dominance effects. However, the approach we propose is general and does not require dominance effects. Indeed, our approach will also work under an additive model because the additive model is a special case of the more general model we use for conditioning. However, if the mode of action of the locus is strictly additive, conditioning on a parental allele dosage may be enough to reduce the bias. Therefore, in future studies, we plan to assess the superiority of conditioning on parental mating types relative to conditioning on allele dosages. Furthermore, we plan to assess the principle we proposed in a broader range of realistic circumstances. We are, particularly, interested in investigating two situations. The first is to evaluate the performance of the proposed approach in a multi-locus context for models involving epistatic interactions, which seem common (Zhu et al., 2015). The second situation is one where there is a selection bias on the exposure, X . Since X is a collider of $G,H,and\;\bar{U}$ , if a subpopulation was sampled according to X (people with X higher than the threshold, for example), spurious correlations among $G,H,and\;\bar{U}$ might occur. In this case, conditioning on parental genetic mating types can account for the correlation between G and H but not for the correlation between $G\;and\;\bar{U}$ because $\bar{U}$ is not a heritable variable.

However, regardless of the limitations suggested previously, conditioning on parental mating types in MR can strengthen assumptions and help avoid type I error inflation and bias, when a heritable confounding variable is associated with the instrumental variable in MR.

## Data Availability

The datasets presented in this study can be found in online repositories. The names of the repository/repositories and accession number(s) can be found in: Zenodo (doi: 10.5281/zenodo.6972710).
